# Towards the Development of an Affordable and Practical Light Attenuation Turbidity Sensor for Remote Near Real-Time Aquatic Monitoring

**DOI:** 10.3390/s20071993

**Published:** 2020-04-02

**Authors:** Jarrod Trevathan, Wayne Read, Simon Schmidtke

**Affiliations:** 1 Institute of Integrated and Intelligent Systems, Griffith University, Nathan, Brisbane, QLD 4111, Australia; w.read@griffith.edu.au; 2 Substation33, Kingston, Logan, QLD 4114, Australia; sschm9@hotmail.com

**Keywords:** environmental monitoring, off-the-shelf technologies, Internet of Underwater Things, turbidity, affordable sensors, calibration

## Abstract

Turbidity is a key environmental parameter that is used in the determination of water quality. The turbidity of a water body gives an indication of how much suspended sediment is present, which directly impacts the clarity of the water (i.e., whether it is cloudy or clear). Various commercial nephelometric and optical approaches and products exist for electronically measuring turbidity. However, most of these approaches are unsuitable or not viable for collecting data remotely. This paper investigates ways for incorporating a turbidity sensor into an existing remote aquatic environmental monitoring platform that delivers data in near real-time (i.e., 15-min intervals). First, we examine whether an off-the-shelf turbidity sensor can be modified to provide remote and accurate turbidity measurements. Next, we present an inexpensive design for a practical light attenuation turbidity sensor. We outline the sensor’s design rationale and how various technical and physical constraints were overcome. The turbidity sensor is calibrated against a commercial turbidimeter using a Formazin standard. Results indicate that the sensor readings are indicative of actual changes in turbidity, and a calibration curve for the sensor could be attained. The turbidity sensor was trialled in different types of water bodies over nine months to determine the system’s robustness and responsiveness to the environment.

## 1. Introduction

Nutrient run-off and sediment deposition are significant factors that influence water quality. The amount of suspended particulate matter in a water body directly impacts the water’s clarity and the biological/chemical processes that occur within the water [1,2]. The amount of suspended sediment can be indicated via *turbidity* [3]. In general, the higher the level of turbidity, the less visibility or light penetration there is through the water column. 

Turbidity is a major determinant of the condition and productivity of an aquatic system. Increased turbidity changes in an ecosystem can significantly reduce the survivability of some plant and fish species [4]. Turbidity also directly impacts the water body’s usability by humans for recreation and safety purposes and can negatively influence the water’s overall aesthetic value. High levels of turbidity can indicate the presence of potentially harmful microorganisms and other contaminants.

Various factors can affect an aquatic environment’s turbidity level. Natural causes include erosion from rivers and streams, flooding events, algae, and decomposition of plant matter (phytoplankton). Human interference factors include nutrient run-off and soil erosion from farming, increased sediment deposition from construction activities, and contamination of ground water tables. Therefore, an inexpensive and accurate way of measuring turbidity in near real-time that is physically easy to use/deploy is highly sought after [5,6,7,8].

Numerous methods exist for measuring turbidity [1,9,10]. Most of these are based on the premise of visual inspection (either electronically or physically) of the passage of light through a solution. In general, the more colloidal materials in the water, the less light penetration there is due to light scattering by and light absorption of the particles. Turbidity measurement devices could be as simple as visually examining a Secchi disk via a turbidity tube [11] or as sophisticated as a nephelometric turbidity measuring device. However, the need exists to find an inexpensive, yet precise way to remotely monitor turbidity in near real-time (e.g., 15-min intervals), to determine how a water body changes during dynamic events such as floods and periods of heavy rainfall.

Turbidimeter designs have been proposed for applications such as quality control for the food industry [12], field measurements of suspended solids [13,14], and dynamic operation of small appliances [15]. Some proposals examine low-cost monitoring of turbidity in drinking water [15,16,17,18]. Some work has been undertaken on low-cost sensor designs [19], distributed real-time turbidity monitoring [20,21], and using machine learning techniques for predicting sediment load in waterways [22,23,24,25,26,27]. However, few designs specifically target the application of cost-effective remote environmental monitoring, nor are commercial turbidity sensors practical for remote in situ deployment over an extended period.

We are working on an initiative to create an affordable, remote aquatic environmental monitoring platform [28,29,30,31,32]. Our work thus far has shown that such an approach is indeed possible with current technologies. The platform measures key environmental parameters via electronic sensors every 15-min and transmits these readings back to an Internet of Things dashboard. The motivation for this paper is to determine whether an off-the-shelf turbidity sensor would be fit for purpose to adapt for use as part of the platform. The platform is designed as a remote early indication system of water quality. Sensors need to be capable enough to inform the end user when a change in conditions is occurring. Detailed scientific measurements should then be undertaken by industrial-grade equipment. 

This paper presents the development and implementation of an affordable and practical light attenuation turbidity sensor. First, we scrutinise a commercially available light attenuation turbidity sensor design and discuss its shortcomings. We then present the rational and design goals for a new turbidity sensor and show how our sensor design was developed for use in the remote aquatic monitoring system. The turbidity sensor is calibrated against and compared to a more expensive nephelometric turbidity sampling device using a Formazin calibration standard. The turbidity sensor has been used in actual field deployments including a dam, creek catchment, river, and urban water bodies.

This paper is structured as follows: Section 2 outlines existing approaches and standards for measuring turbidity. Section 3 investigates a commercial light attenuation turbidity sensor. Section 4 presents the design for a new low-cost turbidity sensor and outlines the challenges that were overcome during its development and calibration. Section 5 describes how the sensor performed in several actual field studies under different circumstances; and Section 6 provides some concluding remarks and avenues for future work.

## 2. Finding a Viable Approach to Electronically Measure Turbidity in Aquatic Environments

### 2.1. Approaches and Standards for Measuring Turbidity 

Turbidity can be measured electronically or manually using various techniques. A common metric for stating turbidity is the *Nephelometric Turbidity Unit* (NTU). The higher the NTU, the more turbid the water is. Table 1 provides typical NTU values for different types of water bodies under natural conditions [4]. Note that drinking water fit for human consumption should ideally be less than 1 NTU (and no greater than 5 NTU) [33,34].

**Figure 1:**
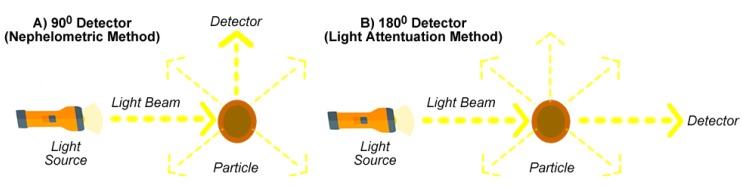
Different methods for measuring the light scattering effects of suspended particles.

There are various techniques for measuring turbidity in a solution. The underlying premise for all techniques is that there is a light source and some form of light detector. The level of turbidity is determined by the transmission and scattering of light by the suspended particles in the solution. Most methods are based on detecting the backscatter of light taken at a 90° angle to the light source (Figure 1A). Various nephelometric water quality standards have been proposed around this approach including the *Environmental Protection Agency* (EPA) Method 180.1, ISO 7027, the Hach Method 10133, and Standard Methods 2130B (see Table 2). These methods define the physical properties to which the sensor must conform and the measurement units. Different backscatter ratios can also be used where the light detector takes samples at different angles to capture additional information depending on the properties of the particle that is causing the backscatter (i.e., larger particles scatter light at smaller angles compared with the way small particles scatter light).

An alternate electronic approach to measuring turbidity is based on *light attenuation* (or transmittance). The light detector is placed at a 180° angle to the light source (Figure 1B). The amount of light that reaches the light detector provides an indication of turbidity. The decrease in light intensity can be due to scattering by particles or from light adsorption from colour [1]. Sensors based on this approach are commonly found in dishwashers and washing machines [35]. No strict standards exist governing the operation of transmittance turbidity sensors, as they are not specifically designed for health-critical water quality applications.

Historically, turbidity was measured according to the now-obsolete *Jackson Turbidity Unit* (JTU). JTU is the inverse measure of the length of a column of water needed to obscure a candle flame completely when viewed through it. The *Formazin Nephelometric Unit* (FNU) indicates that the instrument has measured light scatter at a 90° angle in conformance with the ISO 7027 international standard. NTU is analogous except the standard being adhered to is either the USEPA Method 180.1 or Standard Methods (USA standards). *Formazin Attenuation Units* (FAU) refers to measurements taken from devices employing the light attenuation method where the light scatter is measured at 180° to the light source.

### 2.2. Selecting an Appropriate Viable Turbidity Measurement Approach

Table 3 presents several major commercially available options for measuring turbidity. Cost, interoperability, and inability to be deployed in an ongoing basis in the field rule out most of these options for our platform. Furthermore, the Secchi disk method is a manual process, which makes it unsuitable for our platform. The light attenuation turbidity sensors appear to be the only viable solution.

The TS-300B sensor has low sensitivity (i.e., 0–1000 NTU), so it was ruled out. The TSD-10 sensor did not have a price listed on the manufacturer’s web site. This leaves the DF Robot Gravity analogy turbidity sensor. The Gravity turbidity sensor costs approximately $9.90 U.S. Furthermore, a maximum range of 3000 NTU should make this sensor appropriate for use in most water bodies where our platform will be deployed (refer to Table 1). The challenge for this paper is to establish whether the Gravity turbidity sensor (or a variant thereof) can provide reliability, accuracy, and precision via a 180° angle transmittance measurement, which is comparable to the more expensive 90° angle nephelometric approaches.

## 3. Evaluation of a Commercial Light Attenuation Turbidity Sensor

This section describes the technical detail, behaviour, and calibration of the DF Robot Gravity analogue turbidity sensor, to determine if it can provide rudimentary environmental measurements useful for our platform.

### The DF Robot Gravity Analogue Turbidity Sensor

**Figure 2:**
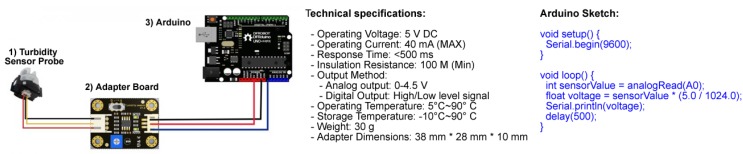
Wiring diagram, setup, and specifications for the DF Robot Gravity analogue turbidity sensor.

Figure 2 depicts the main components and technical specifications for the Gravity turbidity sensor. The sensor consists of: (1) a sensor probe; (2) the adapter board; and (3) an Arduino. The sensor probe houses the electronics for measuring the turbidity level (note that it is not waterproof in its original form). The adapter board contains an operational amplifier (opamp) comparator circuit with a trimpot to adjust the trigger/threshold level for the digital output.

The Arduino supplies the power to the system. The sensor operates at five volts with an analogue value (between 0 and 1023) representing the sensor voltage being returned on the Arduino’s analogue pin (refer to the Arduino sketch).

**Figure 3:**
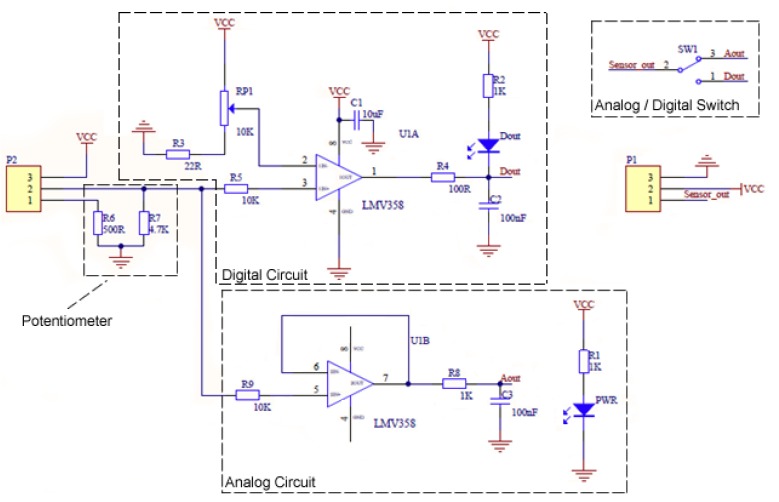
DF Robot Gravity analogue turbidity sensor electronics schematic.

Figure 3 illustrates the Gravity sensor schematic as given by the DF Robot. The sensor can operate in one of two modes: (1) analogue; or (2) digital. In either mode, the sensor operates by sending a signal via an *Infrared* (IR) *Light Emitting Diode* (LED) across a solution to an IR phototransistor. All things being equal, the turbidity is approximated as a function of the reduction in voltage received by the IR phototransistor due to the scattering of IR light by particles (or light adsorption). The digital mode provides a hard threshold value that indicates when there is a distinct change in turbidity (e.g., change versus no change). However, unless the analogue values are understood, there is no certain way of knowing what the digital output measurably means in terms of triggering the NTU threshold. As such, we focus our attention on understanding the sensor’s analogue behaviour.

**Figure 4:**
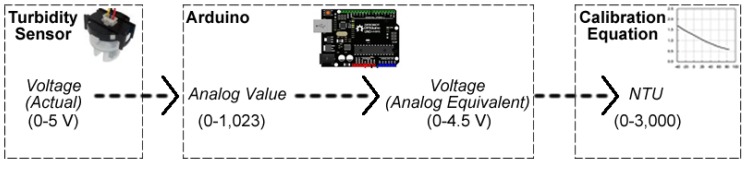
The process involved in converting an analogue sensor reading to a calibrated turbidity value.

Figure 4 illustrates the analogue sensor reading and conversion process (left to right). The sensor outputs the voltage drop between the IR LED and IR phototransistor, which is in the range of 0–5 V. The Arduino measures this via its analogue pin as a value between 0 and 1023. The Arduino converts the analogue value to a voltage value (using the equation sensorValue × (5 V/1024)). Note the maximum value of 4.5 V, which is explained later. The Arduino voltage value is applied to a calibration equation against a known turbidimeter NTU standard to provide an approximate NTU value in the range of 0–3000.

**Figure 5:**
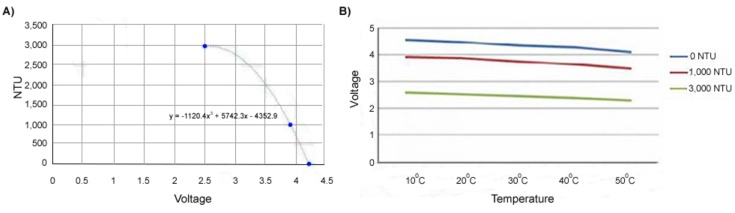
(**A**) The relationship between turbidity and voltage. (**B**) The effect of temperature on sensor readings.

Figure 5A presents a chart by DF Robot illustrating the relationship between voltage and turbidity for the sensor. They state that the sensor will output 4.1 ± 0.3 V in pure water (i.e., NTU < 0.5) when the temperature is 10°~50° C. They also provide the following equation for converting voltage to turbidity:

NTU = −1120.4(*x* ± 0.3)^2^ + 5742.3(*x* ± 0.3) − 4352.9 (1)

At approximately 10° C, the sensor can be expected to provide 4.1 V for 0 NTU, 3.9 V for 1000 NTU, and 2.5 V for 3000 NTU, respectively. Note that there is an inverse relationship between voltage and NTU (i.e., the higher the voltage, the lower the NTU). The sensor is most accurate in the range 0–1000 NTU. Precision tends to deteriorate markedly in the range 1000–3000 NTU (under the calibration curve provided by the vendor).

**Figure 6:**
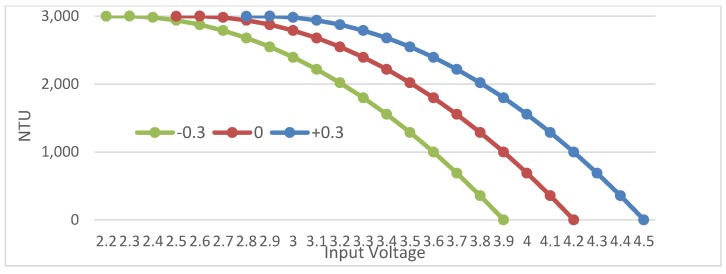
The Gravity turbidity sensor calibration curve with ±0.3 error.

Figure 5B shows the effects of temperature on the voltage output by the sensor and the corresponding NTU values (presented on the DF Robot website). Note that turbidity itself is not affected by temperature. It is the electrical outputs of the sensor that will fluctuate with temperature [1]. As temperature increases, the output of the LED decreases, and less light will reach the IR phototransistor. This introduces about a 0.6 V overall variance across measurements between 10°~50 °C for Gravity turbidity sensors. Figure 6 presents the three calibration curves with zero being the expected and −0.3 and +0.3 being the lower and upper bounds on the respective expected readings.

Note that the sensor also contains a potentiometer that can adjust the amount of power able to go through the LED circuit (between 0 and 5 V). The potentiometer allows the range of results to be increased or decreased to calibrate the sensor against pure water initially. The maximum output of 4.5 V takes into consideration the decrease in voltage when passing through the sensor head’s plastic housing and pure water (i.e., NTU < 0.5).

The specifics of the calibration process undertaken by the vendor are unknown. This includes the calibration method/steps, environmental conditions (temperature and interference from ambient IR light), variations across sensors, and the turbidity device against which it is calibrated (e.g., NTU- or FNU-compliant). As such, an independent recreation of the vendor’s calibration could not be conducted, nor the information provided by the vendor verified.

A ±0.3 V error/margin in readings is an extremely significant range, which makes the accuracy of the vendor’s calibration questionable. This is essentially a 12% variation, which over a 0–3000 NTU range translates to approximately ±150 NTU error. Furthermore, under operational settings, issues such as biofouling over time will impact the sensor’s accuracy. It would be informative to know how much interference can be expected when used in operational settings and for how long the sensor’s readings can be relied on. 

Additionally, the sensor is constructed from a HDPE (*High Density Polyethylene*) turbidity head casing. This substance is extremely difficult to get to bond with other types of plastics and sealants. As such, we simply could not seal the sensor in its current form to even attempt to recreate the vendor’s calibration.

## 4. Development of an Affordable and Practical Light Attenuation Turbidity Sensor

**Figure 7:**
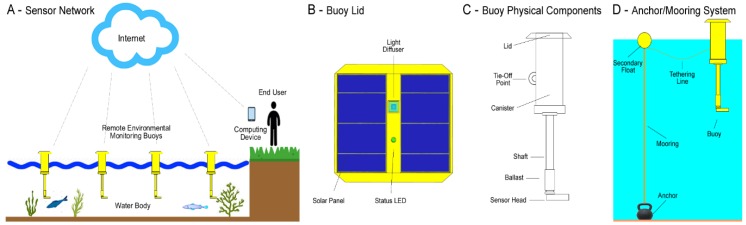
The remote aquatic environmental monitoring system.

Figure 7A illustrates our remote aquatic environmental monitoring system. This section outlines the development of an affordable and practical turbidity sensor for use as part of this platform. We show how the Gravity turbidity sensor’s components were adapted for use in the construction of our turbidity sensor to provide remote, near real-time turbidity readings.

### 4.1. Turbidity Sensor Design

The main electronics for our platform were contained in the buoy’s canister (see Figure 7C). This component was responsible for system control, power management, timing, data logging and telemetry. The second major component was a sensor head that was located 0.5 metres underwater. The sensor head consisted of numerous sensors. For the ease of reference, let us assume for now that the sensor head only contained a light sensor, temperature sensor, and turbidity sensor. 

To incorporate the Gravity turbidity sensor into our aquatic monitoring platform, the following issues needed to be addressed:

1) *Re-engineering of the electronics and integration*: The Gravity turbidity sensor contained additional hardware (i.e., the adaptor board) and functionality (i.e., digital mode) that were not necessary for our aquatic monitoring platform. The turbidity sensor must be powered and controlled by the greater aquatic monitoring platform, seamlessly work in conjunction with other sensors, and its data remotely sent back to a web server.
2) *Water proofing*: The Gravity turbidity sensor head was not waterproof. Therefore, a way to seal the sensor head electronics was required (either via chemical and/or mechanical means).
3) *Calibration*: Calibration was required to determine the sensor’s functioning and turbidity measurements against an industry-accepted standard.

**Figure 8:**
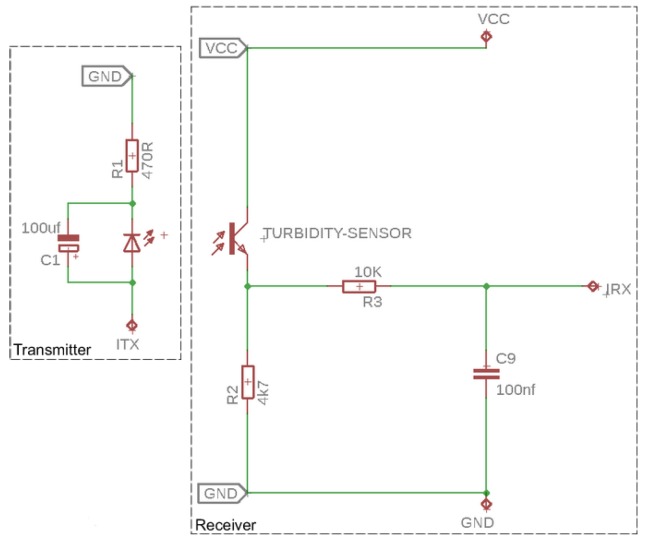
The aquatic monitoring platform turbidity sensor electronics schematic.

The Gravity turbidity sensor contained functionality and hardware that was not necessary for our aquatic monitoring platform. Therefore, we developed a new scaled-back turbidity sensor design. Figure 8 shows the electronics schematic for the turbidity sensor. The circuit retained the original LED and IR phototransistor from the Gravity turbidity sensor. However, all the remaining electronics were reduced to a bare minimum. 

The most significant change was that the Arduino now had direct control over the voltage supplied to the LED (i.e., the potentiometer was no longer required). *Pulse Width Modulation* (PWM) was used to control the power supplied to the IR LED. PWM operates by providing either zero volts or five volts in a cycle; by increasing or decreasing the duration of the five volts over time, the power delivered to the IR could be increased or decreased. Note that PWM was the simplest method of powering the sensor without the requirement of having to add additional electronic components such as an analogue-to-digital convertor.

The left side of Figure 8 depicts the LED transmitter. The aquatic monitoring platform used an Arduino Mega micro controller. The Mega contained fifteen PWM output and runs six internal clocks. We changed the default frequency of 490.20 Hz to 31,372.55 Hz in order to maximise the PWM cycling, thereby providing the most stable voltage. A 100 uF capacitor was used to smooth out the voltage provided to the LED. The capacitor maintained a consistent amount of power in the periods of zero volts during the PWM duty cycle. This formed an aggressive low-pass filter to turn a digital PWM signal essentially into an ongoing stable voltage for the sensor.

The second part of the schematic is the IR phototransistor. A 4.7 K resistor biased the IR phototransistor. An increase of IR light on the base of the IR phototransistor increased the current flowing through it, increasing the output voltage. This was further filtered by a low pass filter (a 10 K resistor and 100 nf capacitor) that blocked high frequency signals from being output.

**Figure 9:**
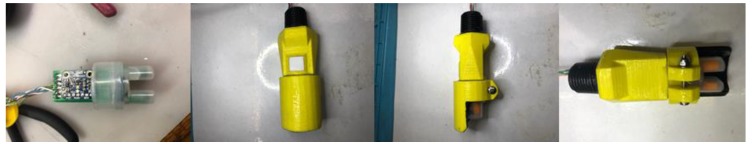
Encasing and waterproofing the turbidity sensor.

The next significant challenge was waterproofing the sensor. In this instance, the turbidity sensor formed part of a sensor head with two other sensors (i.e., light and temperature). The Gravity probe plastic was notoriously difficult to bond with glues and other sealants (it was an HDPE low surface energy plastic). We experimented with epoxy resins to cast the sensor head. However, these impacted the optical properties of the sensor (i.e., they interfered with the IR LED/ IR phototransistor lens). Figure 9 shows the sensor head solution at the time of writing. The LED/ IR phototransistor was plugged into a cavity using hot melt glue. Scotch-Weld^TM^ 8005 was used to attach the HDPE to the *Acrylonitrile Butadiene Styrene* (ABS) printed component. A light shield was then attached around the bonded surface via a 316 stainless steel screw to provide mechanical strength.

### 4.2. Sensor Calibration

In order to get meaningful values from our turbidity sensor, a calibration process must be undertaken. Calibration essentially involved taking multiple readings from one device and comparing those readings with known values (i.e., what the measurements actually should be). Typically, the known values came from readings taken from an already calibrated device.

To undertake calibration, we used a Hach turbidimeter based on the ISO 7027 nephelometric standard for measuring turbidity. Calibration of the Hach was performed by entering calibration mode and reading calibration samples at a known turbidity. We used Formazin calibration samples that approximated several known NTU values (i.e., <0.2, 20, 200, 1000, 4000, and 7500) (note that Formazin is the industry standard for turbidity calibration.)

Ideally, when the Hach is calibrated, it should give exactly the same turbidity every time a water sample verification reading occurs in non-calibration mode. To test this assumption, we calibrated the Hach against the aforementioned Formazin turbidity samples. In non-calibration mode, we took 10 readings of each respective known turbidity sample in order to determine the accuracy of the readings. Table 4 shows the coefficient of variation across the 10 readings for each sample. This allowed us to ascertain that the relative error for the Hach was less than 0.5% (i.e., the Hach would only show at most a 0.5% deviation when reading the same sample).

**Figure 10:**
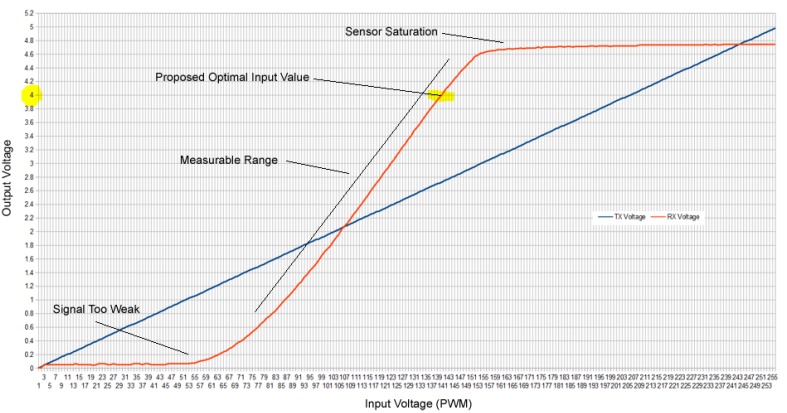
Example electrical response of the sensor to different input pulse width modulation values.

The next step in the process was to calibrate and determine the relative error rate of our turbidity sensor and the variations between sensors. This involved understanding the electrical properties and the standard response of our turbidity sensor across a range of input values. We constructed ten light attenuation turbidity sensors. Figure 10 illustrates a typical response curve for our turbidity sensors. Here, we uniformly input all the PWM values between 0 and 255 to observe the voltage received (referred to as a *PWM sweep*). In this instance, we used tap water (NTU < 1). The sensor was completely shielded from light to prevent interference from ambient IR light. Temperature was also monitored and carefully controlled to ensure that all readings were taken at the same average temperature.

All sensors had a curve similar to the one shown in Figure 10. However, each sensor varied in its power and response. In this example, at PWM values >150, the sensor tended to saturate. PWM values <60 were difficult for the sensor to get any sort of reading. Therefore, the measurable range for the sensor appeared to be PWM values between 60 and 150. The optimal PWM value to use should be somewhere near the top of the curve just before the saturation effect occurs. However, the exact PWM value for each sensor would be different depending on the sensor’s response to differing turbidity values. In contrast to the DF Robot Gravity sensor’s “one size fits all” calibration equation, each sensor needed to be individually calibrated to determine the optimal PWM value.

To observe the impact of temperature on the turbidity sensors, we exposed the sensors to various conditions. Three tests were conducted using tap water. The first control test was done at room temperature (25 °C). The second test involved cooling the sensors uniformly down to approximately −6° to −9 °C and repeating the same process. In the final test, the sensors were heated up to 47° to 52° C. At lower temperatures, the sensors became marginally more responsive, as was expected. At higher temperatures, the sensors became less responsive. However, the actual changes due to temperature were not that significant. For the areas where these sensors were deployed (18°–25 °C), we decided that compensating for temperature was probably not worth the effort, and calibration at a uniform room temperature would be sufficient.

Next, the Formazin samples were diluted by 50% volume with distilled water. This allowed us to essentially double the number of Formazin samples against which to calibrate the 10 sensors. This gave us the following samples: 10, 20, 50, 100, 400, 800, 2000, and 4000 NTU. The PWM sweep was repeated for all 10 sensors across all Formazin samples in controlled lighting/temperature conditions. This was done to establish at which PWM value the sensor responded best to the turbidity sample. 

**Figure 11:**
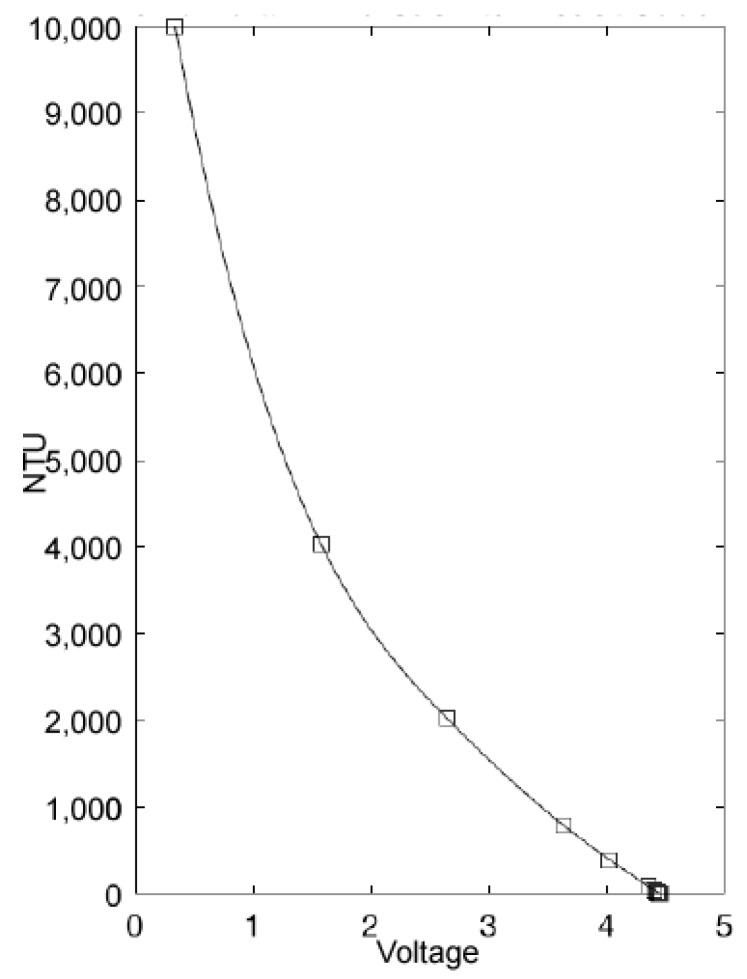
Voltage versus Nephelometric Turbidity Units (NTU): Least squares fit.

Once the PWM value was chosen, the actual calibration curve was determined for each sensor. Figure 11 shows the calibration curve for Sensor 6, which was indicative of the behaviour for all the sensors. The fit for NTU values >100 NTU was very good, indicating that the sensor was extremely reliable and accurate for measuring higher levels of turbidity. This result was significantly more accurate than the calibration equation provided by the DF Robot for their Gravity turbidity sensor. However, the reliability and accuracy decreased somewhat when the samples had turbidities below 100 NTU. We believe that these issues were more due to the small amounts of Formazin in these samples, leading to heterogeneity in the sample and hence inconsistent readings. We also noted that the Hach turbidity sensor showed some variation around these lower level turbidity samples, which supported our conjecture. However, further research and experimentation are required to determine the exact issue. The higher-level calibration readings could be used to estimate the turbidity for values less than 100, and these estimates correlated well with the averaged readings for these samples (<100 NTU). Consequently, we believe the sensor was accurate for all turbidity levels and very accurate for levels in the range of 100 NTU to 4000 NTU.

## 5. Field Deployments

To observe the performance of the turbidity sensor, several deployments were undertaken in various indicative water bodies. Theses water bodies included lakes, creeks, rivers, and dams to gain a holistic perspective of turbidity conditions. Areas where the buoys were deployed included Lake Ellerslie and Slacks Creek in Logan City, Wyaralong Dam in the Scenic Rim and Ross River, Idalia, and Keyatta Lake in Townsville Queensland Australia. The deployment sites were chosen based on existing interest by water authorities/researchers, and independent water quality analyses were taken in parallel. The buoys were deployed over seven months (at the time of writing). Each buoy took turbidity readings at 15-min intervals. These data were remotely sent back and displayed via our dashboard.

**Figure 12:**
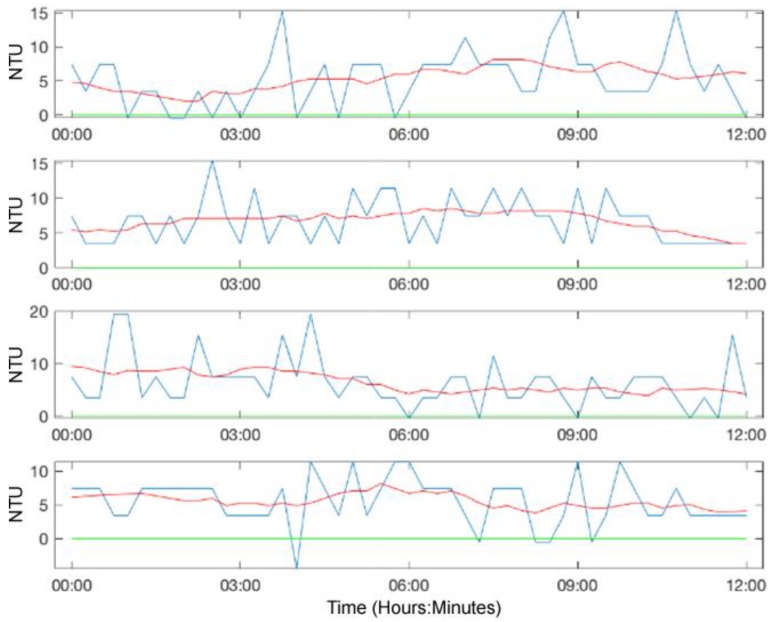
Calibrated turbidity data from the Wyaralong Dam deployment. The plot shows 48 h of turbidity readings taken over four consecutive night time periods of a twelve-hour duration. The blue line displays the raw turbidities, while the red line is a running average of the five previous and post estimates.

The results of these deployments demonstrated that the turbidity sensor was reactive to its environment and indicative of change (Figure 12; the red line is the smoothed turbidity estimates averaged over the five previous and post estimates). For example, in conditions where there were clearly high amounts of suspended sediment, the NTU value for the sensor would read much higher than water bodies with less sediment. We also observed phenomena whereby during notable rainfall events, the NTU value would rise significantly due to an influx of sediment being washed into the system, which would gradually fall again as the sediment settled. 

There was a degree of ambient IR interference during daytime readings. Even though the sensor was shielded from the direct sun, the sensor as picked up refracted light through the water. As such, the night time readings proved to be more stable when this interference was not present. Future work would be to take a sensor reading without the transmitter turned on to capture the amount of ambient IR and then subtract this from the official reading to compensate for the interference.

Different water bodies foul at different rates. Some of the deployments were maintained regularly (i.e., the sensor was cleaned), whereas other locations did not have any maintenance. The impacts of fouling can be seen as the gradual rise in NTU value over time as it became more difficult to transmit signals through the lenses. This evidenced the need for regular maintenance to ensure optimal readings. Future work involves studying the fouling rates of differing water bodies and determining a dynamic calibration adjustment to offset the fouling error. This will allow the sensors to be deployed for longer periods between maintenance cycles.

## 6. Conclusions

This paper presented the development and implementation of an affordable light attenuation turbidity sensor for use as part of a remote, near real-time aquatic monitoring project. Most commercial turbidity sensors are too expensive or are not practical for remote deployment. Light attenuation turbidity sensors appear to be the most economical. Our sensor design was initially adapted from a commercially available light attenuation turbidity sensor. However, the sensor was redesigned for our specific project, resulting in a simpler and more flexible circuit. 

The turbidity sensor was compared to an industry-standard turbidity sampling device. We determined the optimal values to power the sensors based on each sensor’s individual electrical response. We then used Formazin turbidity standards to calibrate the sensors. Compared to the original commercial vendor’s information, we determined that the sensor could accurately and reliably measure turbidity between 100 and 4,000 NTU. The sensor was indicative of a change in turbidity conditions. However, further work is required to determine what the calibration issue was with values below 100 NTU. At this stage, we suspect that Formazin at lower NTU introduced the problem of the non-homogeneity of the particles in the water, which made it difficult to detect them. Future work involves investigating whether a new microcontroller could be used to provide higher resolution for the sensor readings. For example, the ESP32 microcontroller has a 12 bit resolution on its input pins, which means the sensor reading range extends to 4096 values (compared to 1024 for the Arduino).

The turbidity sensor was trialled in actual field deployments across a range of differing types of water bodies. These deployments clearly showed that the sensor was reactive to dynamic conditions. During the deployments, ambient IR interference proved to be an issue during the day. In future work, we will compensate for light interference by taking an ambient IR reading prior to a turbidity reading. This will allow the light interference to be subtracted from the actual turbidity reading. Altering the circuitry to include a digital-to-analogue convertor will facilitate the interleaving of readings.

When deployed in the environment, fouling has a gradual impact on the sensor over time. The fouling impacts the amount of light being picked up by the IR receiver as it reduces the transparency of the HDPE casing. As such, a regular maintenance regime must be in place. This involves giving the sensor a quick wipe to remove any fouling build up. Future work involves applying dynamic calibration adjustments to combat fouling and other interference. That is, once the fouling rate is known, it can be compensated at the server level for the error. This will increase the amount of time the sensor can be deployed before any manual human intervention.

Additionally, it would be useful to explore whether a low-cost nephelometric turbidity sensor could be developed (i.e., where the light detector is at 90° to the light source) [42]. 

## Figures and Tables

**Table 1:** Typical turbidity values for a range of different water bodies.

Water Body	NTU	Location
Upland rivers and lowland rivers	1–50	Most upland streams have low turbidity. High values may be observed during high flow events. Turbidity in lowland rivers can be extremely variable. Values at the low end of the range are found in rivers flowing through well-vegetated catchments and at low flows. Values at the high end of the range are found in rivers draining slightly disturbed catchments and in many rivers at high flows.
Lakes and reservoirs/wetlands	1–100	Most deep lakes and reservoirs have low turbidity. However, shallow lakes and reservoirs may have higher natural turbidity due to wind-induced re-suspension of sediments. Lakes and reservoirs in catchments with highly dispersible soils will have high turbidity.
Estuarine and marine	0.5–10	Low turbidity values are normally found in offshore waters. Higher values may be found in estuaries or inshore coastal waters due to wind-induced re-suspension or to the input of turbid water from the catchment.

**Table 2:** Turbidity measurement techniques.

Type/Method	Light Source	Method
EPA Method 180.1	Tungsten lamp with a colour temperature of 2000 K–3000 K.	Nephelometric technology that measures light scatter at a 90° angle from the light path.
ISO 7027	Monochromatic light source at a wavelength of 860 nm, with a spectral bandwidth of 60 nm.	Requirement of the primary photo detector angle of 90° ± 2.5 degrees.
Hach Method 10133	Laser diode red light, with a wavelength of 630 nm	Nephelometric technology that measures light scatter at a 90° angle from the light path.
Standard Methods 2130B	Tungsten-filament lamp light source with a colour temperature of 2,200–3,000 K.	Requirement that the photo detector be centred at 90°.
Light Attenuation	Typically infrared light sources.	The loss of light between a light source and a detector directly across from it (180°).
Turbidity Tube	Indirect sunlight/daylight.	Adding sample water into a tube with the back turned to toward the Sun, taking the reading until the disc at the bottom is no longer visible.

**Table 3:** A cost comparison of turbidity measurement devices.

Name	Brand	Price (USD)	Range	Compliance
LPV442.99.03022 [36]	Hach	$8,549	0–1000 NTU	Hach Method 10133
HI88713-01 [37]	Hanna Instruments	$1,690	0.00 to 4000 NTU (ratio mode) 0.00 to 1000 NTU (non-ratio mode)	ISO 7027
HI88703-01 [38]	Hanna Instruments	$1,640	0.00 to 4000 NTU (ratio mode) 0.00 to 40.0 NTU (non-ratio mode)	Standard Methods 2130B EPA Method 180.1
SE_TurbTube	Select Scientific	$70	10–400 NTU	Secchi Disk Method
SEN0189 Gravity [39]	DF Robot	$9.90	0–3000 NTU	Light Attenuation Method
TSD-10 [40]	Amphenol	Unavailable	0–4000 NTU	Light Attenuation Method
TS-300B [41]	CTS Corporation	$2.69	0–1000 NTU	Light Attenuation Method

**Table 4:** Relative error rates of Formazin samples taken by the Hach.

Sample Type	<0.2 NTU	20 NTU	200 NTU	1000 NTU	4000 NTU	7500 NTU
Formazin	7.4	0.53	0.63	0.43	0.19	0.07

## References

[1] Website: Fondriest Environmental. Measuring Turbidity, TSS, and Water Clarity. https://www.fondriest.com/environmental-measurements/equipment/measuring-water-quality/turbidity-sensors-meters-and-methods.

[2] Website: Geoscience Australia (n.d.). Turbidity. Retrieved from OzCoasts. http://www.ozcoasts.gov.au/indicators/turbidity.jsp.

[3] Taylor R.E., Bull D.W. (1998). Turbidity Sensor. U.S. Patent.

[4] Website: Australian and New Zealand Environment and Conservation Council Guidelines for Fresh and Marine Water Quality. www.waterquality.gov.au/guidelines/anz-fresh-marine.

[5] Orwin J.F., Smart C.C. (2005). An Inexpensive Turbidimeter for Monitoring Suspended Sediment. Geomorphology.

[6] Lambrou T.P., Panayiotou C.G., Anastasiou C.C. A Low-Cost System for Real Time Monitoring and Assessment of Potable Water Quality at Consumer Sites. Proceedings of the Sensors IEEE.

[7] Kelley C.D., Krolick A., Brunner L., Burklund A., Kahn D., Ball W.P., Weber-Shirk M. (2014). An Affordable Open-Source Turbidimeter. Sensors.

[8] Wijenayake N.A.J., Alahakoon P.M.K. Development of a Cost-Effective Turbidimeter. Proceedings of the Water Professionals’ Day Symposium.

[9] ISO/IEC 7027:1999 Water Quality–Determination of Turbidity. https://www.iso.org/obp/ui/#iso:std:iso:7027:ed-3:v1:en.

[10] O’Dell J.W. (1993). Method 180.1: Determination of Turbidity by Nephelometry. Environmental Monitoring Systems Laboratory Office of Research and Development.

[11] Swift T.J., Perez-Losada J., Schladow S.G., Reuter J.E., Jassby A.D., Goldman C.R. (2006). Water clarity modeling in Lake Tahoe: Linking suspended matter characteristics to Secchi depth. Aquat. Sci..

[12] Novo C., Bilro L., Ferreira R., Alberto N., ANTUnes P., Leitão C., Pinto J.L. Plastic Optical Fibre Sensor for Quality Control in Food Industry. Proceedings of the Fifth European Workshop on Optical Fibre Sensors.

[13] Bilro L., Prats S., Pinto J.L., Keizer J.J., Nogueira R.N. Turbidity Sensor for Determination of Concentration, Ash Presence and Particle Diameter of Sediment Suspensions. Proceedings of the 21st International Conference on Optical Fibre Sensors.

[14] Bilro L., Alberto N., Pinto J.L., Nogueira R. (2012). Optical Sensors Based on Plastic Fibers. Sensors.

[15] Omar A.F., MatJafri M.Z. (2011). The Swift Turbidity Marker. Phys. Educ..

[16] Lambrou T.P., Anastasiou C.C., Panayiotou C.G. (2009). A Nephelometric Turbidity System for Monitoring Residential Drinking Water Quality, Sensor Applications, Experimentation, and Logistics.

[17] Liu Y., Xu H. Design of a MCU-controlled Laser Liquid Turbidimeter Based on OPT101. Proceedings of the International Conference on Optical Instrumentation and Technology, International Society for Optics and Photonics.

[18] Pereira J.D., Postolache O., Girao P.S., Ramos H. SDI-12 Based Turbidity Measurement System with Field Calibration Capability. Proceedings of the IEEE Canadian Conference on Electrical and Computer Engineering.

[19] Garcia A., Pérez M.A., Ortega G.J.G., Dizy J.T. (2007). A New Design of Low-Cost Four-Beam Turbidimeter by Using Optical Fibers. IEEE Trans. Instrum. Meas..

[20] Tai H., Li D., Wang C., Ding Q., Wang C., Liu S. (2012). Design and Characterization of a Smart Turbidity Transducer for Distributed Measurement System. Sens. Actuators A Phys..

[21] Mylvaganam S., Jakobsen T. Turbidity Sensor for Underwater Applications, Sensor Design and System Performance with Calibration Results. Proceedings of the OCEANS’98 Conference Proceedings (IEEE).

[22] Olyaie E., Banejad H., Chau K.W., Melesse A.M. (2015). A comparison of various artificial intelligence approaches performance for estimating suspended sediment load of river systems: A case study in United States. Environ. Monit. Assess..

[23] Alizadeh M.J., Nodoushan E.J., Kalarestaghi N., Chau K.W. (2017). Toward multi-day-ahead forecasting of suspended sediment concentration using ensemble models. Environ. Sci. Pollut. Res..

[24] Alizadeh M.J., Kavianpour M.R., Danesh M., Adolf J., Shamshirband S., Chau K.W. (2018). Effect of river flow on the quality of estuarine and coastal waters using machine learning models. Eng. Appl. Comput. Fluid Mech..

[25] Chen X.Y., Chau K.W. (2019). Uncertainty Analysis on Hybrid Double Feedforward Neural Network Model for Sediment Load Estimation with LUBE Method. Water Resour. Manag..

[26] Shamshirband S., Jafari Nodoushan E., Adolf J.E., Abdul Manaf A., Mosavi A., Chau K.W. (2019). Ensemble models with uncertainty analysis for multi-day ahead forecasting of chlorophyll a concentration in coastal waters. Eng. Appl. Comput. Fluid Mech..

[27] Kargar K., Samadianfard S., Parsa J., Nabipour N., Shamshirband S., Mosavi A., Chau K.W. (2020). Estimating longitudinal dispersion coefficient in natural streams using empirical models and machine learning algorithms. Eng. Appl. Comput. Fluid Mech..

[28] Trevathan J., Atkinson I., Read W., Bajema N., Lee Y.J., Scarr A., Johnstone R. Developing low-cost intelligent wireless sensor networks for aquatic environments. Proceedings of the Sixth International Conference on Intelligent Sensors, Sensor Networks and Information Processing (ISSNIP).

[29] Trevathan J., Johnstone R., Chiffings T., Atkinson I., Bergmann N., Read W., Stevens T. (2012). SEMAT—the next generation of inexpensive marine environmental monitoring and measurement systems. Sensors.

[30] Lee Y.J., Trevathan J., Atkinson I., Read W. (2015). The Integration, Analysis and Visualization of Sensor Data from Dispersed Wireless Sensor Network Systems Using the SWE Framework. J. Telecommun. Inf. Technol..

[31] Trevathan J., Hamilton L., Read W. (2016). Allocating Sensor Network Resources Using An Auction-Based Protocol. J. Theor. Appl. Electron. Commer. Res..

[32] Trevathan J., Johnstone R. (2018). Smart Environmental Monitoring and Assessment Technologies (SEMAT)—A New Paradigm for Low-Cost, Remote Aquatic Environmental Monitoring. Sensors.

[33] World Health Organization (2004). Guidelines for Drinking-Water Quality.

[34] Dearmont D., McCarl B.A., Deborah A. (1998). Tolman. Costs of water treatment due to diminished water quality: A case study. Water Resour. Res..

[35] Smith J.M., Schneider D.A., Dausch M.E., Whipple W. (1996). III Dishwasher with Turbidity Sensing Mechanism. U.S. Patent.

[36] Website: Hach. www.hach.com/tu5200-laboratory-laser-turbidimeter-with-rfid-iso-version/product?id=27464937041.

[37] Website: Hanna Instruments. ISO 7027 Compliant Benchtop Turbidity Meter. Hannainst.com/hi88713-iso-turbidity-benchtop-meter.html.

[38] Website: Hanna Instruments. EPA Compliant Benchtop Turbidity Meter. Hannainst.com/hi88703-turbidity-benchtop-meter.html.

[39] Website: DF Robot. Gravity: Analog Turbidity Sensor for Arduino. www.dfrobot.com/product-1394.html.

[40] Website: Amphenol. https://au.mouser.com/new/Amphenol/GE-NovaSensor-Turbidity/.

[41] Website: InnovatorsGuru. TS-300B|High Quality Arduino Turbidity Sensor. https://www.innovatorsguru.com/ts-300b-arduino-turbidity-sensor/.

[42] Kitchener B.G., Dixon S.D., Howarth K.O., Parsons A.J., Wainwright J., Bateman M.D., Cooper J.R., Hargrave G.K., Long E.J., Hewett C.J. (2019). A low-cost bench-top research device for turbidity measurement by radially distributed illumination intensity sensing at multiple wavelengths. HardwareX.

